# Risk prediction for *Staphylococcus aureus* surgical site infection following cardiothoracic surgery; A secondary analysis of the V710-P003 trial

**DOI:** 10.1371/journal.pone.0193445

**Published:** 2018-03-21

**Authors:** Fleur P. Paling, Karina Olsen, Kristin Ohneberg, Martin Wolkewitz, Vance G. Fowler, Mark J. DiNubile, Hasan S. Jafri, Frangiscos Sifakis, Marc J. M. Bonten, Stephan J. Harbarth, Jan A. J. W. Kluytmans

**Affiliations:** 1 Julius Center for Health Sciences and Primary Care, University Medical Center Utrecht, Utrecht, Netherlands; 2 Institute for Medical Biometry and Statistics, University Medical Center Freiburg, Freiburg, Germany; 3 Division of Infectious Diseases, Duke University Medical Center, Durham, North Carolina, United States of America; 4 Merck & Company Incorporation, Kenilworth, New Jersey, United States of America; 5 MedImmune, Gaithersburg, Maryland, United States of America; 6 AstraZeneca Pharmaceuticals LP, Gaithersburg, Maryland, United States of America; 7 Department of Medical Microbiology, University Medical Center Utrecht, Utrecht, Netherlands; 8 Geneva University Hospitals and Medical School, Geneva, Switzerland; 9 Amphia Hospital, Breda, The Netherlands; Chang Gung Memorial Hospital, TAIWAN

## Abstract

**Background:**

Identifying patients undergoing cardiothoracic surgery at high risk of *Staphylococcus aureus* surgical site infection (SSI) is a prerequisite for implementing effective preventive interventions. The objective of this study was to develop a risk prediction model for *S*. *aureus* SSI or bacteremia after cardiothoracic surgery based on pre-operative variables.

**Materials/Methods:**

Data from the Merck Phase IIb/III *S*. *aureus* vaccine (V710-P003) clinical trial were analyzed. In this randomized placebo-controlled trial, the effect of preoperative vaccination against *S*. *aureus* was investigated in patients undergoing cardiothoracic surgery. The primary outcome was deep/superficial *S*. *aureus* SSI or *S*. *aureus* bacteremia through day 90 after surgery. Performance, calibration, and discrimination of the final model were assessed.

**Results:**

Overall 164 out of 7,647 included patients (2.1%) developed *S*. *aureus* infection (149 SSI, 15 bacteremia, 28 both). Independent risk factors for developing the primary outcome were pre-operative colonization with *S*. *aureus* (OR 3.08, 95% confidence interval [CI] 2.23–4.22), diabetes mellitus (OR 1.87, 95% CI 1.34–2.60), BMI (OR 1.02 per kg/m^2^, 95% CI 0.99–1.05), and CABG (OR 2.67, 95% CI 1.91–3.78). Although vaccination had a significant (albeit modest) protective effect, it was omitted from the model because its addition did not significantly change the coefficients of the final model and V710-vaccine development has been discontinued due to insufficient efficacy. The final prediction model had moderate discriminative accuracy (AUC-value, 0.72).

**Conclusion:**

Pre-operative *S*. *aureus* colonization status, diabetes mellitus, BMI, and type of surgical procedure moderately predicted the risk of *S*. *aureus* SSI and/or bacteremia among patients undergoing cardiothoracic surgery.

## Introduction

Surgical site infection (SSI) with or without bacteremia is a common post-operative complication responsible for increased morbidity, mortality, and health care costs[1–3]. The most important cause of SSIs among patients undergoing clean surgery is *Staphylococcus aureus* [4–6] which frequently colonizes the nares and skin in the healthy population. In preoperative patients, carriage is associated with an elevated risk for post-operative SSI and bacteremia [7,8]. Yet the ability to identify preoperative patients at highest risk for *S*. *aureus* SSI or post-operative bacteremia is inadequate [9]. As preemptive pathogen-specific preventive interventions are under development, it is important to reliably identify those patients at substantial risk for this complication [10].

For this study, data from the Merck Phase IIb/III *S*. *aureus* vaccine study (V710-P003) were analyzed [11]. This double-blinded, randomized, placebo-controlled trial investigated the effect of a pre-operative vaccine targeting *S*. *aureus* on the incidence of postoperative *S*. *aureus* bacteremia and/or deep sternal wound infection in adult patients undergoing cardiothoracic surgery through postoperative day 90. V710 was not sufficiently efficacious in preventing the primary endpoint by prespecified criteria, and overall mortality rates for the placebo or vaccine group were not significantly different. The trial was stopped prematurely after interim analysis showed lack of efficacy as well as a numerically higher mortality rate in the subset of vaccine recipients developing *S*. *aureus* infections. Pre-operative *S*. *aureus* colonization status was documented as part of protocol-stipulated procedures.

In the current *post hoc* analysis of the prospectively collected data from this clinical trial, we aimed to develop a pathogen-specific risk prediction model for *S*. *aureus* SSI and/or bacteremia in patients after cardiothoracic surgery based on information ascertainable preoperatively.

## Materials and methods

Data from the randomized, double-blind, placebo-controlled trial of Merck Phase IIb/III *S*. *aureus* vaccine (V710-P003, registered at clinicaltrials.gov under the identifier NCT00518687) were used for this *post hoc* analysis [11]. Because the clinical trial was stopped in part due to unacceptably low vaccine efficacy, we included both placebo and vaccine recipients in this analysis. Data were available on all efficacy outcomes. Decolonization procedures and pre-operative surgical prophylaxis were provided according to local standards of care for the international sites participating in the trial. However, decolonization methods were neither mandated by protocol nor routinely recorded. The original study protocol was approved by the institutional review boards or ethical review committees at each site and executed in accordance with Good Clinical Practice guidelines.

### Patient population

Adult patients undergoing elective cardiothoracic surgery were eligible for inclusion. Exclusion criteria, described in more detail elsewhere, included active infection, pregnancy, and immunosuppression[11,12].

### Primary outcome

The primary outcome was a binary (yes/no) composite endpoint through day 90 after surgery, which included at least one of the following *S*. *aureus* diagnoses: deep/superficial sternal wound infection (including mediastinitis), deep/superficial harvest site infection, and bacteremia (defined as at least one positive blood culture growing *S*. *aureus*). All cases were adjudicated by an independent committee using diagnostic criteria established by the Center for Disease Control and Prevention (CDC) [13].

### Potential predictors and their management

A list of candidate predictors was defined prior to initiating this analysis, based on clinical judgment and availability in the database, including pre-operative *S*. *aureus* colonization status, pre-operative antibiotic use, diabetes mellitus, type of cardiothoracic procedure, body mass index (BMI), age, and sex.

We defined a patient to be colonized if nasal *S*. *aureus* carriage was documented by culture at any moment before surgery. This assumption was chosen because literature indicates that colonization status is largely dependent on the patient’s constitution and thus relatively constant over time[7].

Pre-operative antibiotic use was defined as any systemic antibiotic use within 6 months before surgery, excluding pre-operative prophylaxis. A timeframe of 6 months pre-operatively was chosen, considering that previous studies had shown that the microbiome can be affected after antibiotic usage for this period of time[14]. Diabetes mellitus was coded as yes if there was a confirmed diagnosis of diabetes mellitus, regardless of duration of disease or need for diabetic agents. Gestational diabetes was not included. Surgical procedure type was dichotomized to coronary artery bypass grafting (CABG) or not. The combination of CABG and cardiac valve surgery was coded as CABG. Cardiac valve surgery alone or other cardiothoracic surgery types including median sternotomy were coded as ‘no CABG’.

Age and BMI were used as continuous variables; it was checked whether fractional polynomials improved model performance[15]. Missing values (n = 152) of *S*. *aureus* colonization status were imputed using multiple imputation techniques[16].

Univariate logistic regression analysis was performed on the mentioned variables. Variables with a univariate p≤0.157 were entered into the final multivariable model, roughly corresponding to the selection threshold based on the Akaike information criterion when considering p-values [17]. Tests of interactions between pre-operative *S*. *aureus* colonization status and BMI or diabetes mellitus were performed (p-value<0.05).

### Regression model and model performance

A logistic regression model was fitted with the variables described above. Overall model performance was assessed by measuring the explained variation (Nagelkerke R^2^)[18]. Calibration of the model was assessed by plotting the observed proportion of events against the predicted risks for groups defined by ranges of individual predicted risks. For the assessment of the discrimination of the model, a receiver operating characteristic (ROC) curve was plotted and the area under the curve (AUC or c-statistic) was computed. Internal validation was assessed by performing 200 bootstrap samples.

### Sensitivity analyses

#### Competing events

Patients might have died within 90 days post-surgery without reaching the primary outcome, which means that death is a competing event for the primary outcome. As a sensitivity analysis, a Fine & Gray model was fitted to account for the time-to-event, considering death as a competing event [19]. Subdistribution hazard ratios for SSI were calculated as an alternative measure (by acknowledging the time-dependency) for the odds ratios. Cumulative incidence functions were calculated with stratification by risk score groups using the Aalen-Johansen estimator[20].

#### Vaccine effect

Considering that we used a slightly different primary outcome compared to the initial study (originally superficial or harvest site infections were not included), it was assessed whether a vaccine-effect was present (p-value <0.05) and whether adding vaccination to the model significantly altered the effect estimates.

All statistical analyses were performed using R version 2.10.00. [21]

## Results

In the final analysis, 7,647 patients were included. Their baseline characteristics are described in Table 1. Overall 165 out of 7,647 included patients (2.1%) developed *S*. *aureus* SSI and/or bloodstream infection, including 122 (1.6%) patients with SSI without bacteremia, 28 (0.4%) patients with bacteremic SSI, and 15 patients (0.2%) with post-operative bacteremia without SSI.

**Table 1:** Baseline characteristics.

	With outcome N = 165	Without outcome N = 7,482	Total N = 7,647
**Age (years)**	64.9 (10.8)	63.9 (12.4)	**63.9 (12.4)**
**Gender:** female	53 (30.0)	2,467 (33.0)	**2,520 (33.0)**
**Pre-operative *S*. *aureus* colonization**	67 (42.0)	1,364 (18.2)	**1,431 (18.7)**
**BMI (kg/M**^**2**^**)**	29.0 (5.7)	27.6 (5.3)	**27.6 (5.3)**
**Diabetes mellitus**	71 (45.3)	1,765 (23.6)	**1836 (24.0)**
**Pre-operative antibiotic use**	10 (5.3)	653 (8.7)	**663 (8.7)**
**CABG**	113 (68.5)	3,075 (41.1)	**3,188 (41.7)**
**Vaccination**	66 (40.1)	3,747 (50.0)	**3,813 (49.9)**
Death*	7 (4.2)	229 (3.1)	**236 (3.1)**

Values are given as means (SD), and numbers (%). SD = standard deviation, BMI = body mass index, CABG = coronary artery bypass grafting

*Death within 90 days post-surgery

### Predictors of *S*. *aureus* SSI and/or bacteremia

Several pre-operative variables were univariately associated with the primary outcome: pre-operative colonization status with *S*. *aureus* (OR 3.07, 95% confidence interval [CI] 2.23–4.20), diabetes mellitus (OR 2.45, 95% CI 1.78–3.34), CABG (OR 3.01, 95% CI 2.24–4.35), and BMI (OR 1.04 per kg/m^2^ increase, 95% CI 1.02–1.07). No significant interaction was found between pre-operative *S*. *aureus* colonization and either BMI or diabetes mellitus (p-values 0.196 and 0.089, respectively).

Independent risk factors identified during multivariate analysis were pre-operative colonization status (OR 3.08, 95% CI 2.23–4.22), diabetes mellitus (OR 1.87, 95% CI 1.34–2.60), CABG (OR 2.67, 95% CI 1.91–3.78) and BMI (OR 1.02 per unit increase, 95% CI 0.99–1.05) (Table 2).

**Table 2:** Univariate and multivariate logistic regression analysis.

	Unadjusted OR (95% CI)	p-value	Adjusted OR (95% CI)	p-value
**Age**^**1**^	1.01 (0.99–1.02)	0.315	Not included	
**Gender: female**^**2**^	0.96 (0.69–1.33)	0.818	Not included	
**Pre-operative *S*.*aureus* colonization**	3.01 (2.23–4.20)	<0.001*	3.08 (2.23–4.22)	<0.001*
**BMI**^**1**^	1.04 (1.02–1.07)	0.001*	1.02 (0.99–1.05)	0.148
**Diabetes mellitus**	2.45 (1.78–3.34)	<0.001*	1.87 (1.34–2.60)	<0.001*
**Pre-operative antibiotic use**	0.67 (0.33–1.22)	0.231	Not included	
**CABG**	3.10 (2.24–4.35)	<0.001*	2.67 (1.91–3.78)	<0.001*
**Vaccination**	0.67 (0.48–0.91)	0.011*	0.67 (0.48–0.91)	0.012*

* Significant at the 0.05 level. OR = odds ratio

1) OR per year of age or kg/M^2^ increase

2) Male is reference category

### Model performance

The mean explained variation of the model as indicated by the Nagelkerke R^2^ was 0.08. The distribution of predicted risks for the event of interest was highly skewed to the left, with more patients in the low risk categories than in the high-risk categories. Only 8.2% of the patients had a risk of ≥5%. Of the 209 *S*. *aureus* colonized, diabetic patients undergoing CABG (i.e. who had all three major risk factors), the risk of developing the event was 11% (n = 23). Of the 3012 patients without any preoperative risk factor, 28 (0.9%) developed the event.

**Fig 1:**
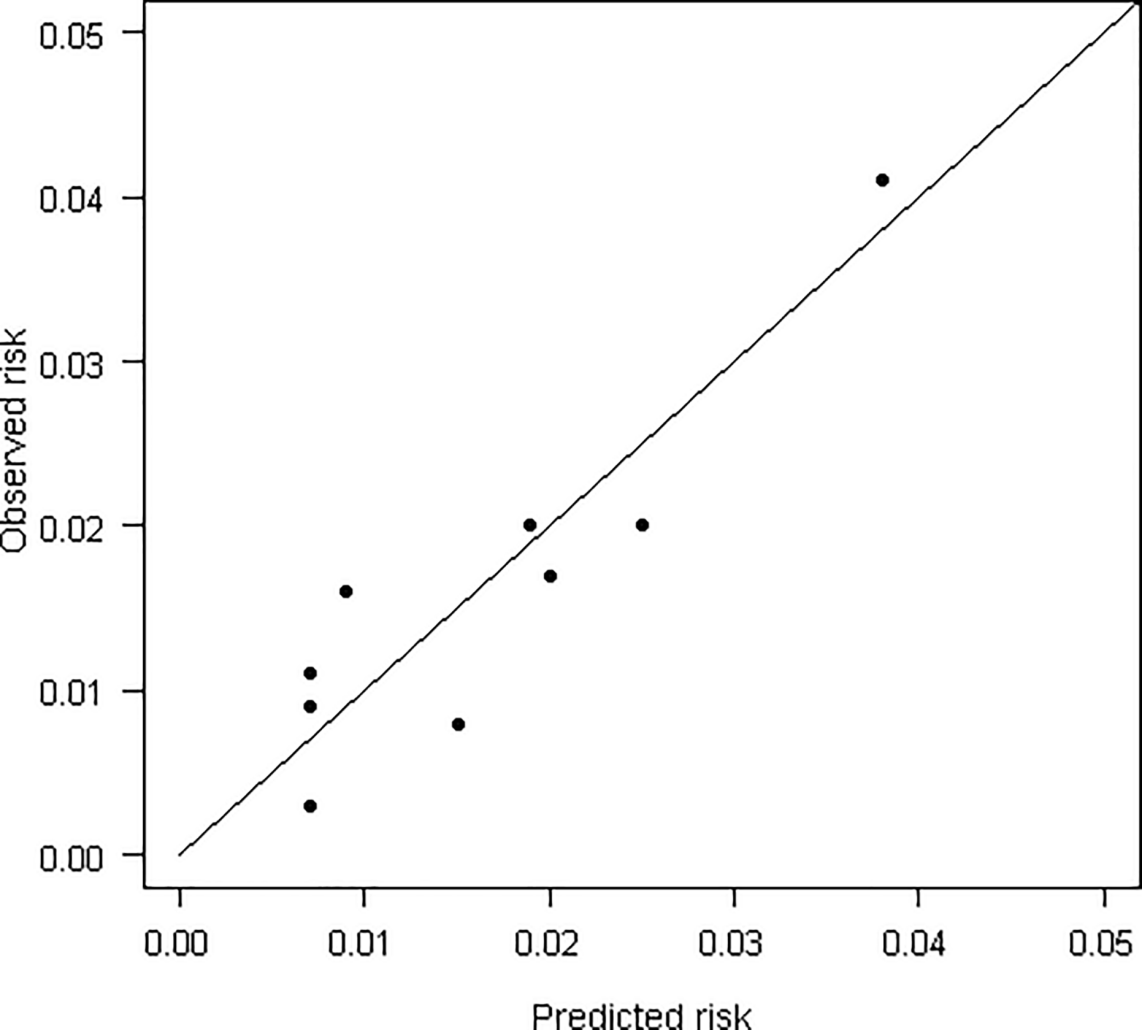
Calibration plot of final model, showing observed risks vs. predicted risks on the primary outcome.

**Fig 2:**
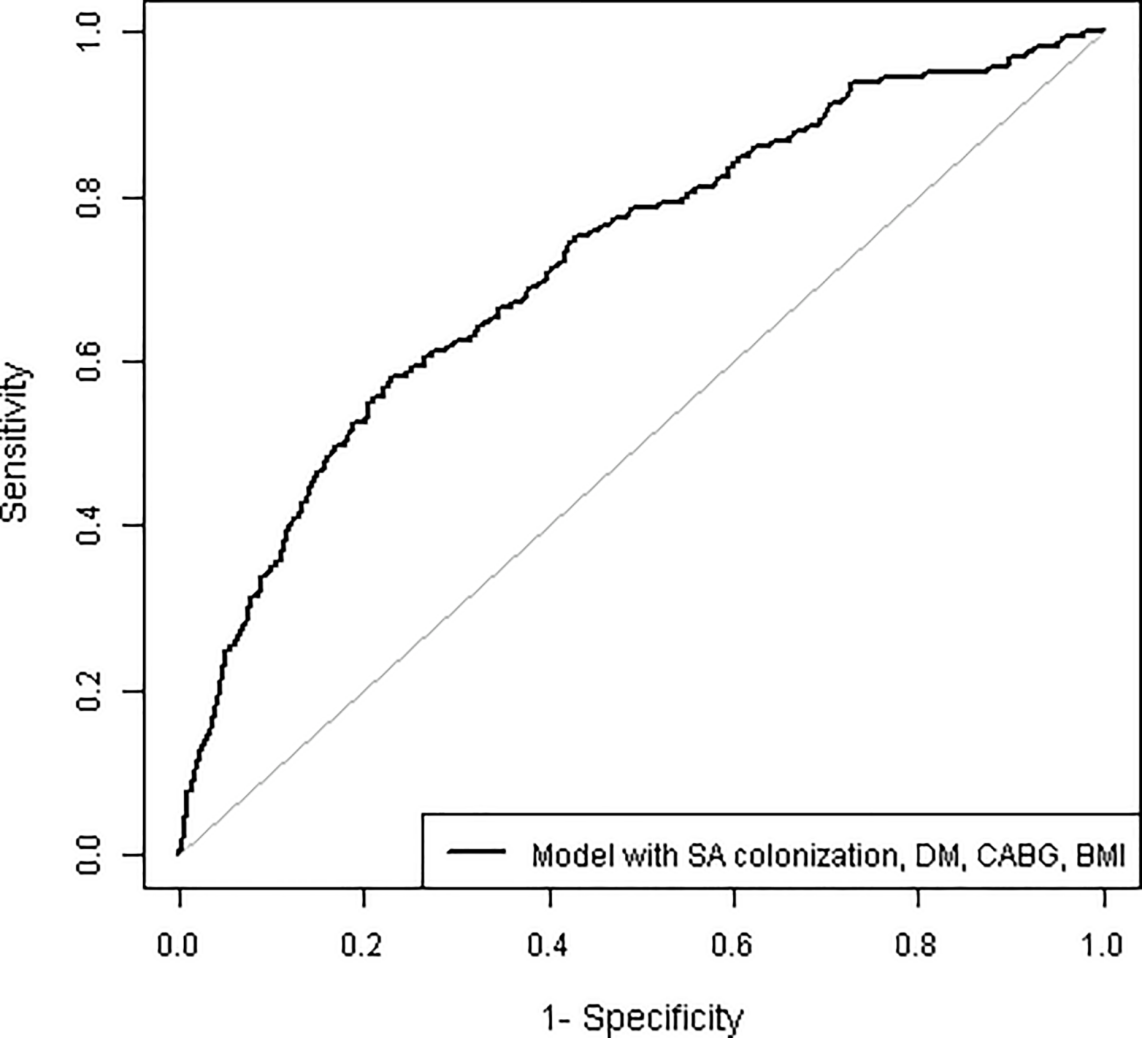
ROC curve of final model, with an AUC of 0.72 (95% CI 0.68–0.76).

**Fig 3:**
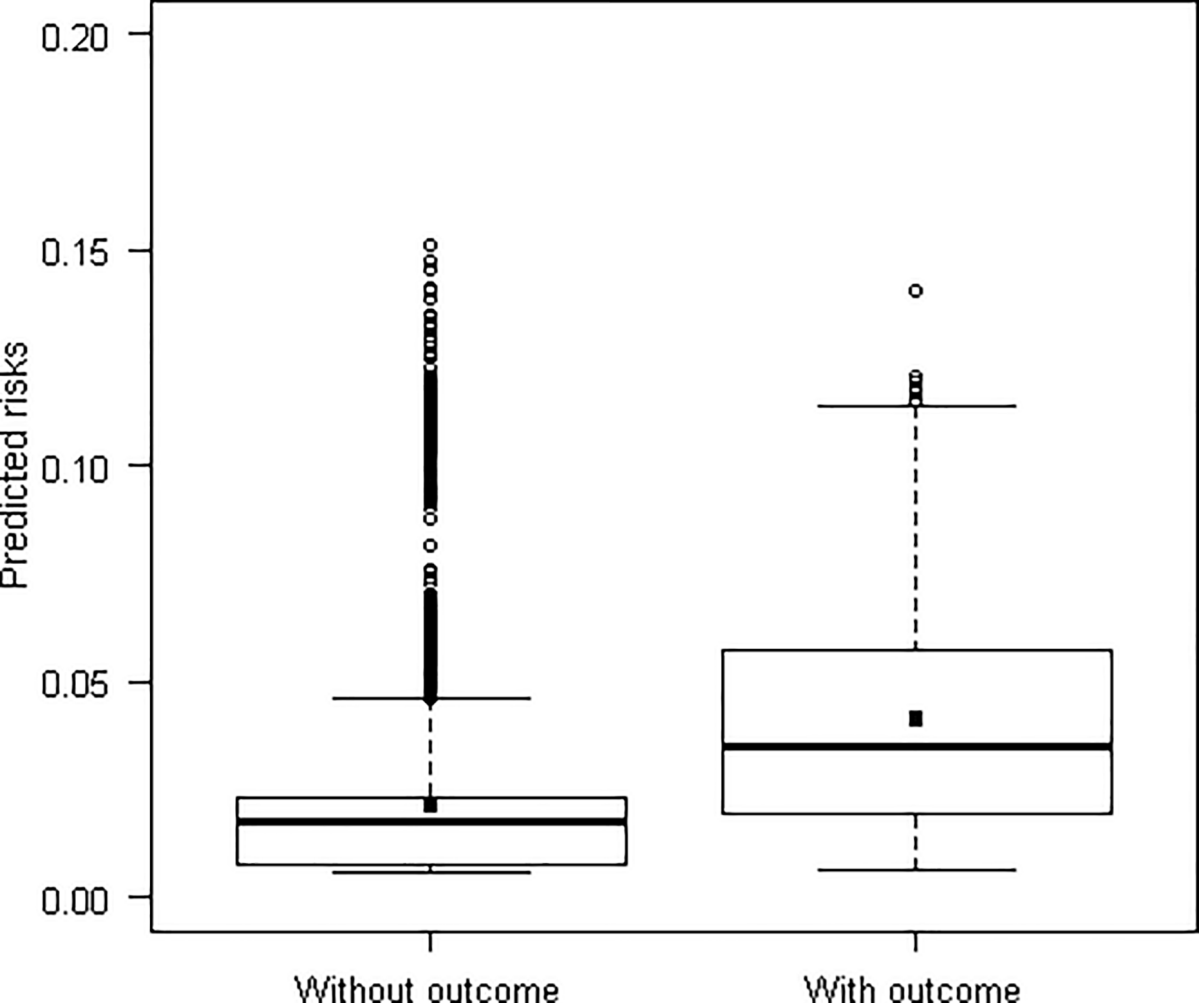
Boxplot showing distribution of predicted risks stratified for groups with/without primary outcome.

Fig 1 shows a calibration plot with average agreement between the observed events and the predicted risks by ranges of individual predicted risks (Hosmer-Lemeshow χ2 = 13.0, p = 0.11). Discrimination of the model was average, with an area under the ROC curve of 0.72 (95% CI 0.68–0.76) (Figs 2 and 3).

### Internal validation

The stability of the final model was further assessed in 200 bootstrap samples. Using these samples, we derived an R^2^ of 0.07 and AUC of 0.72 after correction for optimism. The Somers’ Dxy rank correlation between predicted probabilities and observed responses was 0.43 (0 indicating completely random predictions and 1 indicating perfect predictions).

### Sensitivity analysis

#### Competing risks

A total number of 236 patients died within 90 days post-surgery. Of these, 229 had not yet developed the primary event of interest. Using the Fine & Gray competing risks analysis to assess whether the subdistribution hazard ratios differ from the odds ratios from the logistic regression model, the estimates did not change significantly (maximum observed change was 2%). Hence, the effect of death as a competing risk can largely be ignored.

#### Vaccine effect

Vaccination was univariately associated with the primary outcome. V710 was protective against *S*. *aureus* infection (OR 0.67, 95% CI 0.48–0.91, p = 0.011), and remained so after correction for other predictors (OR 0.67, 95% CI 0.48–0.91, p = 0.012). However, other predictor estimates did not change significantly after incorporating vaccination status, indicating a lack of confounding effect. Furthermore, because the development of this specific vaccine has been discontinued, vaccination was not included as a predictor in the final model.

## Discussion

In this analysis, we built a risk prediction model to determine which preoperative characteristics put patients at higher risk of developing *S*. *aureus* SSI and/or bacteremia after cardiothoracic surgery. We identified *S*. *aureus* colonization, diabetes, increasing BMI, and CABG surgery as independent risk factors. The final prediction model using these readily available predictors performed satisfactorily.

As the frequency and impact of post-surgical infections remain substantial, the relevance of an accurate prediction model remains. Many previous studies have developed and validated risk prediction tools for all-cause surgical site infection in cardiothoracic patients, some of which are frequently used in practice [22,23]. However, practical pathogen-specific models for postoperative *S*. *aureus* infections are scarce. Pathogen-specific prediction may be preferable, anticipating the arrival of targeted preventive measures in the near future [10,24–26]. Furthermore, patients suffering from *S*. *aureus* infections are at substantial risk for bad outcomes and incur higher health care costs[27–30]. This prediction model advances existing literature because it employs simple predictors routinely available in the preoperative patient. The risk difference between a patient not having any risk factor compared with one that has three is 10.1% (0.9% vs. 11.0%). However, in this derivation set, even though the predictors frequently occurred independently of each other, there were only 209 patients (2.7%) having all three factors, still leaving many patients at low or intermediate risk. A previous study by Kanafani et al. showed similar results [9]. Better discrimination between infected and non-infected patients is required to identify a larger patient group that would benefit from new interventions. Comprehensive prospective studies will be required, such as the prospective cohort study called ASPIRE-SSI (Advanced Understanding of Staphylococcus Aureus Infections in Europe—Surgical Site Infections), which is part of the COMBACTE-NET initiative[31,32]. This study will describe risk factors for *S*. *aureus* SSI of approximately 5000 patients across Europe undergoing different types of surgery and is currently ongoing.

A possible option for new model developers could be to use an established, validated prediction score like Euroscore and assess whether adding pathogen-specific variables like colonization status can make the model pathogen-specific[33]. This could have wider implications, considering that implementation would not require any major change in routine practice, should the new prediction model be successful. The recently published ‘*Global guidelines on the prevention of surgical site infection’* specifically stress the need for such a simple, inexpensive screening process, considering that in low- and middle-income countries the logistical and financial burdens that come with a screening and decolonization intervention may be too burdensome to implement on all preoperative patients [34].

A major strength of the current study is the size of the study and the number of participating countries/centers. Furthermore, data collection and patient follow-up was stipulated by protocol and closely monitored, minimizing the amount of missing data during follow-up, and ensuring a high proportion of patients screened for *S*. *aureus* colonization unlikely to occur outside the setting of a clinical trial. Last, but not least, the statistical analyses performed here, including the sensitivity analyses taking into account competing risks were sophisticated and comprehensive.

There are several limitations to this analysis. First of all, decolonization strategies for *S*. *aureus* were neither standardized nor documented. Decolonization methods were likely applied to colonized patients at a majority of the sites [35]. If indeed accurate, this practice would decrease the difference in incidence rate of the primary outcome between colonized and non-colonized patients, as decolonization reduces infection rates in carriers [36,37].

Furthermore, in this study only nares were screened for *S*. *aureus* colonization, thus, carriage on skin or at other sites may have been missed. In other words, there is potential misclassification bias, since some of the “non-colonized” patients may have been colonized elsewhere. This misclassification likely would be independent of *S*. *aureus* bacteremia and SSI, giving rise to a non-differential misclassification of the *S*. *aureus* carrier status. The non-differential misclassification may have biased our estimates towards the null and reduced the discriminative effect of the new prediction model.

Despite the limitations described above, the model performed moderately well. In its present form it may only be useful to indicate an especially high risk for patients having all three risk factors. For subtler prediction and external validation, further enhancement of the model is necessary.

## Conclusion

From this analysis, we can conclude that pre-operative *S*. *aureus* colonization gives a 3x higher OR for *S*. *aureus* SSI / bacteremia in the unsubstantiated (but likely) presence of decolonization procedures. Without decolonization, the risk is likely to be higher. This model that included colonization status, diabetes, and CABG had overall average performance.

## Supporting information

S1 File Full list of approving ethics committees. (DOC)
