# Supplementary material for: Risk prediction for Staphylococcus aureus surgical site infection following cardiothoracic surgery; A secondary analysis of the V710-P003 trial
Source: PLoS One. 2018 Mar 21;13(3):e0193445. doi: 10.1371/journal.pone.0193445 (PMC5862433; doi:10.1371/journal.pone.0193445)
Supplement: S1 File — (DOC) [file pone.0193445.s001.doc]

**S1: Full list of approving ethics committees**

|  |  |
| --- | --- |
|  | **Number of** |
| **Name and Address** |
| **Independent Ethics** | **Subjects** |
| **Committee (IEC)** | **Randomized** |
| Christiana Care | 19 |
| Institutional Review |  |
| Board |  |
| 4735 Ogletown-Stanton |  |
| Rd., MAP 2, Suite 2114 |  |
| Newark, DE 19713 |  |
|  |  |
| Western Institutional | 47 |
| Review Board |  |
| 3535 Seventh Ave. SW |  |
| Olympia, WA 98502 |  |
|  |  |
| MedStar Research | 20 |
| Institutional Review |  |
| Board |  |
| 6495 New Hampshire |  |
| Ave., Suite 201 |  |
| Hyattsville, MD 20783 |  |
|  |  |
| Western Institutional | 35 |
| Review Board |  |
| 3535 Seventh Ave. SW |  |
| Olympia, WA 98502 |  |
|  |  |
|  |  |
| Western Institutional | 42 |
| Review Board |  |
| 3535 Seventh Ave. SW |  |
| Olympia, WA 98502 |  |
|  |  |
| HCA Richmond Division | 29 |
| Institutional Review |  |
| Board |  |
| 2621 Grove Avenue |  |
| Richmond, VA 23220 |  |
|  |  |
|  |  |
|  |  |


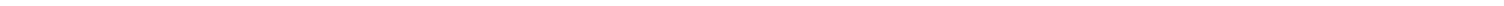

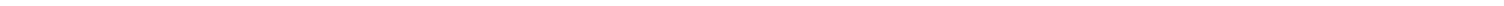


|  |  |
| --- | --- |
|  | **Number of** |
| **Name and Address** |
| **Independent Ethics** | **Subjects** |
| **Committee (IEC)** | **Randomized** |
| Vanderbilt University | 6 |
| Medical Center |  |
| 1313 21st Avenue South |  |
| 504 Oxford House |  |
| Nashville, TN 37232 |  |
|  |  |
| Western Institutional | 17 |
| Review Board |  |
| 3535 Seventh Ave. SW |  |
| Olympia, WA 98502 |  |
|  |  |
| Baptist Hospital | 0 |
| 8900 N. Kendall Drive |  |
| Miami, FL 33176 |  |
|  |  |
|  |  |
|  |  |
| St. Francis Hospital | 0 |
| Institutional Review |  |
| Board |  |
| 100 Port Washington |  |
| Blvd. |  |
| Roslyn, NY 11576 |  |
|  |  |
|  |  |
| University Of Maryland | 94 |
| School Of Medicine |  |
| 685 West Baltimore |  |
| Street |  |
| Baltimore, MD 21201 |  |
|  |  |
|  |  |
|  |  |


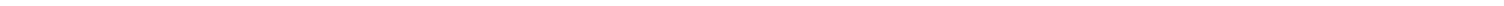

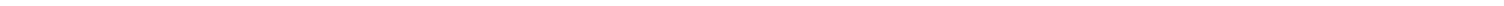

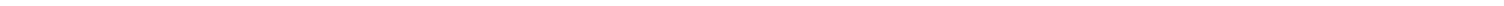


|  |  |
| --- | --- |
|  | **Number of** |
| **Name and Address** |
| **Independent Ethics** | **Subjects** |
| **Committee (IEC)** | **Randomized** |
| Combined Institutional | 0 |
| Review Board |  |
| 555 South 70th Street |  |
| Lincoln, NE 68510 |  |
|  |  |
| Western Institutional | 42 |
| Review Board |  |
| 3535 Seventh Ave. SW |  |
| Olympia, WA 98502 |  |
|  |  |
| Western Institutional | 0 |
| Review Board |  |
| 3535 Seventh Ave. SW |  |
| Olympia, WA 98502 |  |
|  |  |
|  |  |
| Medical College of | 0 |
| Georgia |  |
| Human Assurance |  |
| Committee IRB |  |
| 1120 Fifteenth Street |  |
| Augusta, GA 30912 |  |
|  |  |
|  |  |
| North Texas Institutional | 0 |
| Review Board at Medical |  |
| City |  |
| 7777 Forrest Lane, C-740 |  |
| Dallas, TX 75230 |  |
|  |  |
|  |  |
| Rockingham Memorial | 0 |
| Hospital |  |
| Investigational Review |  |
| Board |  |
| 235 Cantrell Avenue |  |
| Harrisonburg, VA 22801 |  |
|  |  |
| MedStar Health Research | 22 |
| Institute IRB |  |
| 6525 Belcrest Road |  |
| Suite 700 |  |
| Hyattsville, MD 20782 |  |
|  |  |


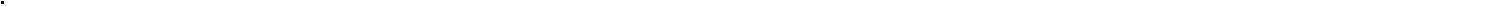


|  |  |
| --- | --- |
|  | **Number of** |
| **Name and Address** |
| **Independent Ethics** | **Subjects** |
| **Committee (IEC)** | **Randomized** |
| Eastern Idaho Regional | 34 |
| Medical Center IRB |  |
| 3100 Channing Way |  |
| Idaho Falls, ID 83404 |  |
|  |  |
|  |  |
| Human Research | 8 |
| Protection Program |  |
| University of Kansas |  |
| Medical Center |  |
| 3901 Rainbow Blvd. |  |
| Kansas City, KS 66160 |  |
|  |  |
| St. Patrick Hospital | 34 |
| Community Medical |  |
| Center, Inc. |  |
| Joint Investigational |  |
| Review Board |  |
| 500 West Broadway |  |
| Missoula, MT 59802 |  |
|  |  |
| Western Institutional | 133 |
| Review Board |  |
| 3535 Seventh Ave. SW |  |
| Olympia, WA 98502 |  |
|  |  |
| Committee on Research | 1 |
| Involving Human |  |
| Subjects |  |
| SUNY |  |
| W-5530 Melville Library |  |
| Stony Brook, NY 11794 |  |
|  |  |
| Advocate Health Care | 5 |
| Institutional Review |  |
| Board |  |
| 205 West Touhy Avenue |  |
| Suite 203 |  |
| Park Ridge, IL 60068 |  |
|  |  |
| St. Vincents Hospital IRB | 133 |
| 8402 Harcourt Road |  |
| Suite 208 |  |
| Indianapolis, IN 46260 |  |
|  |  |


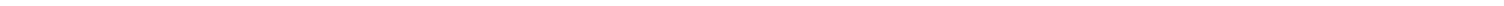


|  |  |
| --- | --- |
|  | **Number of** |
| **Name and Address** |
| **Independent Ethics** | **Subjects** |
| **Committee (IEC)** | **Randomized** |
| Washington Regional | 17 |
| Medical Center |  |
| 3215 N. North Hills Blvd. |  |
| Fayetteville, AR 72703 |  |
|  |  |
| Western Institutional | 31 |
| Review Board |  |
| 3535 Seventh Ave. SW |  |
| Olympia, WA 98502 |  |
|  |  |
| Lehigh Valley Hospital | 30 |
| 17th and Chew Streets, |  |
| 6th Floor |  |
| Allentown, PA 18103 |  |
|  |  |
| Duke Medical Center | 29 |
| Hock Plaza |  |
| 2424 Erwin Road |  |
| Durham, NC 27710 |  |
|  |  |
| Comitato Etico | 1 |
| dell’Azienda Ospedaliera |  |
| San Camillo Forlanini |  |
| Circonvallazione |  |
| Gianicolense, 87 |  |
| Roma 00152 |  |
| Italy |  |
|  |  |
|  |  |
|  |  |
| Comitato Etico | 40 |
| dell’Università Cattolica |  |
| del Sacro Cuore - |  |
| Policlinico Universitario |  |
| Agostino Gemelli |  |
| Largo A. Gemelli, 8 |  |
| Roma 00168 |  |
| Italy |  |
|  |  |
|  |  |


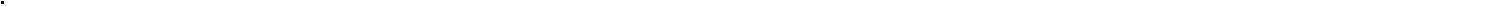


|  |  |
| --- | --- |
|  | **Number of** |
| **Name and Address** |
| **Independent Ethics** | **Subjects** |
| **Committee (IEC)** | **Randomized** |
| Comitato Etico | 47 |
| dell’Università Campus |  |
| Bio-Medico di Roma |  |
| Via Alvaro del Portillo, |  |
| 21 |  |
| Roma 00128 |  |
| Italy |  |
|  |  |
|  |  |
| St. Luke’s Hospital IRB | 81 |
| 4401 Wornall Road |  |
| Kansas City, MO 64111 |  |
|  |  |
| Universitair Ziekenhuis | 155 |
| Gasthuisberg - Cardiale |  |
| Heelkunde |  |
| Herestraat 49 |  |
| 3000 Leuven |  |
| Belgium |  |
|  |  |
|  |  |
| National Taiwan | 94 |
| University Hospital, |  |
| Ethics Review Committee |  |
| No. 7, Chung-Shan South |  |
| Road |  |
| 100 Taipei |  |
| Taiwan |  |
|  |  |
| Royal Free Hospital & | 84 |
| Medical School Research |  |
| Ethics Committee |  |
| Pond Street |  |
| London, England NW3 |  |
| 2QG United Kingdom |  |
| **The research ethics** |  |
| **committee changed its** |  |
| **name during the course** |  |
| **of the study to:** |  |
| NRES Committee |  |
| London – Hampstead |  |
| Nothwick Park Hospital |  |
| Watford Road |  |
| Harrow Middlesex |  |
| HA1 3UJ |  |
| United Kingdom |  |
|  |  |

|  |  |
| --- | --- |
|  | **Number of** |
| **Name and Address** |
| **Independent Ethics** | **Subjects** |
| **Committee (IEC)** | **Randomized** |
| Royal Free Hospital & | 73 |
| Medical School Research |  |
| Ethics Committee |  |
| Pond Street |  |
| London, England NW3 |  |
| 2QG United Kingdom |  |
| **The research ethics** |  |
| **committee changed its** |  |
| **name during the course** |  |
| **of the study to:** |  |
| NRES Committee |  |
| London – Hampstead |  |
| Nothwick Park Hospital |  |
| Watford Road |  |
| Harrow Middlesex |  |
| HA1 3UJ |  |
| United Kingdom |  |
|  |  |
| Royal Free Hospital & | 30 |
| Medical School Research |  |
| Ethics Committee |  |
| Pond Street |  |
| London, England NW3 |  |
| 2QG United Kingdom |  |
| **The research ethics** |  |
| **committee changed its** |  |
| **name during the course** |  |
| **of the study to:** |  |
| NRES Committee |  |
| London – Hampstead |  |
| Nothwick Park Hospital |  |
| Watford Road |  |
| Harrow Middlesex |  |
| HA1 3UJ |  |
| United Kingdom |  |
|  |  |
| NRES Committee | 50 |
| London – Hampstead |  |
| Nothwick Park Hospital |  |
| Watford Road |  |
| Harrow Middlesex |  |
| HA1 3UJ |  |
| United Kingdom |  |

|  |  |
| --- | --- |
|  | **Number of** |
| **Name and Address** |
| **Independent Ethics** | **Subjects** |
| **Committee (IEC)** | **Randomized** |
| Comité de Protection des | 39 |
| Personnes Ile de France |  |
| III |  |
| Hôpital Tarnier Cochin |  |
| 89, rue d’Assas |  |
| 75006 Paris |  |
| France |  |
|  |  |
| Comité de Protection des | 102 |
| Personnes Ile de France |  |
| III |  |
| Hôpital Tarnier Cochin |  |
| 89, rue d’Assas |  |
| 75006 Paris |  |
| France |  |
|  |  |
|  |  |
| Comité de Protection des | 1 |
| Personnes Ile de France |  |
| III |  |
| Hôpital Tarnier Cochin |  |
| 89, rue d’Assas |  |
| 75006 Paris |  |
| France |  |
|  |  |
| Comité de Protection des | 149 |
| Personnes Ile de France |  |
| III |  |
| Hôpital Tarnier Cochin |  |
| 89, rue d’Assas |  |
| 75006 Paris |  |
| France |  |
|  |  |


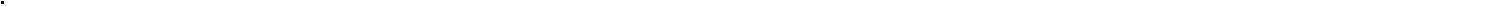


|  |  |
| --- | --- |
|  | **Number of** |
| **Name and Address** |
| **Independent Ethics** | **Subjects** |
| **Committee (IEC)** | **Randomized** |
| Comité de Protection des | 60 |
| Personnes Ile de France |  |
| III |  |
| Hôpital Tarnier Cochin |  |
| 89, rue d’Assas |  |
| 75006 Paris |  |
| France |  |
|  |  |
| Comité de Protection des | 65 |
| Personnes Ile de France |  |
| III |  |
| Hôpital Tarnier Cochin |  |
| 89, rue d’Assas |  |
| 75006 Paris |  |
| France |  |
|  |  |
| Asan Medical Center IRB | 59 |
| 88 Ollimpingno43gil |  |
| Songpa-gu |  |
| Seoul, 138-736 |  |
| Korea |  |
|  |  |
| Samsung Medical Center | 71 |
| IRB |  |
| 50 Irwon-dong |  |
| Kangnam-gu |  |
| Seoul, 135-710 |  |
| Korea |  |
|  |  |
| Institutional Review | 91 |
| Board Severance Hospital |  |
| (250 Seongsan-ro) |  |
| 134 Sinchon-dong |  |
| Seodaemun-gu, Seoul |  |
| 120-752 |  |
| Korea |  |
|  |  |
| Comité de Etica en | 213 |
| Investigación - HNGAI |  |
| Av. Grau 800 La |  |
| Victoria, |  |
| Lima 13 |  |
| Peru |  |
|  |  |
|  |  |


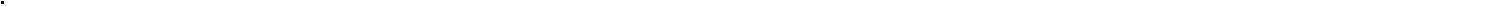

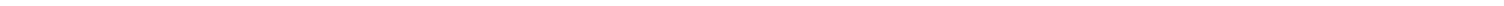


|  |  |
| --- | --- |
|  | **Number of** |
| **Name and Address** |
| **Independent Ethics** | **Subjects** |
| **Committee (IEC)** | **Randomized** |
| Institutional Review | 19 |
| Board - HN Edgardo |  |
| Rebagliati Martins |  |
| Av. Edgardo Rebagliati |  |
| 490 Jesús María |  |
| Lima 11 |  |
| Peru |  |
|  |  |
| Instituto Nacional de | 209 |
| Cardiología Ignacio |  |
| Chávez |  |
| Juan Badiano # 1 |  |
| Colonia Sector 16 |  |
| Tlalpan |  |
| México City 14080 |  |
| Mexico |  |
|  |  |
| Comissão de Ética para |  |
| Análise de Projetos de |  |
| Pesquisa- CAPPesq |  |
| Rua Ovídeo Pires de |  |
| Campos, s/n Prédio da | 97 |
| Administração 5 andar |  |
| sala 105 |  |
| São Paulo – SP |  |
| Brazil |  |
|  |  |
| Comitê de Ética em |  |
| Pesquisa do IDPC |  |
| Av. Dante Pazzanese, 500 | 164 |
| São Paulo – SP |
|  |
| Brazil |  |
|  |  |
| Comitê de Ética do |  |
| Hospital São Lucas - |  |
| PUC | 38 |
| Av. Ipiranga, 6690 |
|  |
| Porto Alegre – RS |  |
| Brazil |  |
|  |  |
| Comitê de Ética em |  |
| Pesquisa em Seres |  |
| Humanos da PUC- |  |
| Campinas |  |
| Rodovia Dom Pedro | 64 |
| I,Km 136 – Parque das |  |
| Universidades – |  |
| Campinas – SP |  |
| Brazil |  |
|  |  |


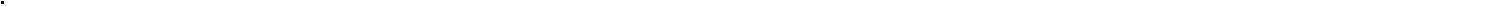

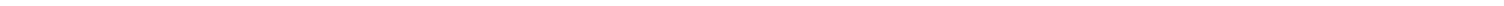


|  |  |
| --- | --- |
|  | **Number of** |
| **Name and Address** |
| **Independent Ethics** | **Subjects** |
| **Committee (IEC)** | **Randomized** |
| Comitê de Ética do |  |
| Hospital Santo Antonio |  |
| Av. Bonfim, 161 | 21 |
| Largo de Roma |
| Salvador – BA |  |
| Brazil |  |
|  |  |
| Comité de Etica | 358 |
| Independiente Latin |  |
| Ethics |  |
| 14 calle 7-13 Z 9 |  |
| Ed.Torre Blanca 10 Nivel |  |
| Guatemala 01015 |  |
| Guatemala |  |
| Sosiaali- ja | 335 |
| terveysministeriö Etene / |  |
| Tukija |  |
| Kirkkokatu 14 PL 33 |  |
| Valtioneuvosto |  |
| Helsinki 00023 |  |
| Finland |  |
| Ethik-Kommission an der | 317 |
| Universität Regensburg |  |
| Franz-Josef-Strauss Allee |  |
| 11 |  |
| Regensburg 93042 |  |
| Germany |  |
|  |  |
| Ethik-Kommission an der | 26 |
| Universität Regensburg |  |
| Franz-Josef-Strauss Allee |  |
| 11 |  |
| Regensburg 93042 |  |
| Germany |  |
|  |  |
| Ethik-Kommission an der | 269 |
| Universität Regensburg |  |
| Franz-Josef-Strauss Allee |  |
| 11 |  |
| Regensburg 93042 |  |
| Germany |  |
|  |  |

|  |  |
| --- | --- |
|  | **Number of** |
| **Name and Address** |
| **Independent Ethics** | **Subjects** |
| **Committee (IEC)** | **Randomized** |
| Ethik-Kommission an der | 0 |
| Universität Regensburg |  |
| Franz-Josef-Strauss Allee |  |
| 11 |  |
| Regensburg 93042 |  |
| Germany |  |
|  |  |
| Ethik-Kommission an der | 13 |
| Universität Regensburg |  |
| Franz-Josef-Strauss Allee |  |
| 11 |  |
| Regensburg 93042 |  |
| Germany |  |
|  |  |
| Ethik-Kommission an der | 235 |
| Universität Regensburg |  |
| Franz-Josef-Strauss Allee |  |
| 11 |  |
| Regensburg 93042 |  |
| Germany |  |
|  |  |
| CEIC Área 7 Hospital | 60 |
| Clínico San Carlos |  |
| Farmacología Clínica, |  |
| Planta 1ª |  |
| Madrid 28040 |  |
| Spain |  |
|  |  |
| CEIC Hospital Clinic i | 29 |
| Provincial |  |
| Villarroel, 170 |  |
| Barcelona 08036 |  |
| Spain |  |
|  |  |
|  |  |
|  |  |
|  |  |
|  |  |


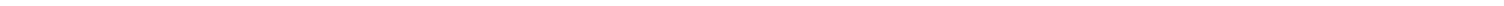


|  |  |
| --- | --- |
|  | **Number of** |
| **Name and Address** |
| **Independent Ethics** | **Subjects** |
| **Committee (IEC)** | **Randomized** |
| CEIC Hospitals Vall | 20 |
| d’Hebron Ed Institut de |  |
| Recerca |  |
| 2ª Pta Secretaria del |  |
| CEIC Passeig Vall |  |
| D’Hebrón, 119-129 |  |
| Barcelona 08035 |  |
| Spain |  |
|  |  |
| CEIC Comunidad | 154 |
| Autónoma del País Vasco |  |
| Edif.Dirección de |  |
| Farmacia C/ Donostia - |  |
| San Sebastián, 1 Vitoria, |  |
| Alava 01010 |  |
| Spain |  |
|  |  |
| CEIC Área 2 H. U. | 206 |
| Princesa |  |
| Diego de Leon, 62 |  |
| Madrid 28006 |  |
| Spain |  |
|  |  |
| CEIC Hospital Clínico | 106 |
| Universitario de Valencia |  |
| Pabellón de Docencia |  |
| Avda. Blasco Ibañez, 17 |  |
| Pab. B - 1ª Planta |  |
| Valencia 46010 |  |
| Spain |  |
|  |  |
| Ethikkommission der | 70 |
| Stadt Wien GF |  |
| Gesundheitswesen |  |
| u.Soziales (MA15) |  |
| TownTown |  |
| Thomas-Klestil-Platz 8 |  |
| Wien 1030 |  |
| Austria |  |
|  |  |
| Western Institutional | 28 |
| Review Board |  |
| 3535 Seventh Ave. SW |  |
| Olympia, WA 98502 |  |
|  |  |
|  |  |
|  |  |
|  |  |

|  |  |
| --- | --- |
|  | **Number of** |
| **Name and Address** |
| **Independent Ethics** | **Subjects** |
| **Committee (IEC)** | **Randomized** |
| Regionala | 91 |
| Etikprövningsnämnden i |  |
| Göteborg |  |
| Box 401 |  |
| 405 30 Göteborg |  |
| Sweden |  |
|  |  |
| Regionala | 54 |
| Etikprövningsnämnden i |  |
| Göteborg |  |
| Box 401 |  |
| 405 30 Göteborg |  |
| Sweden |  |
|  |  |
| Regionala | 25 |
| Etikprövningsnämnden i |  |
| Göteborg |  |
| Box 401 |  |
| 405 30 Göteborg |  |
| Sweden |  |
|  |  |
| St. Luke’s Hospital | 47 |
| 801 Ostrum Street |  |
| Bethlehem, PA 18015 |  |
|  |  |
| Unidad de Investigación | 2 |
| en Salud |  |
| Trasviña y Retes #1317 |  |
| Col. San Felipe |  |
| Chihuahua 31205 |  |
| Mexico |  |
|  |  |
|  |  |
| Regionala | 81 |
| Etikprövningsnämnden i |  |
| Göteborg |  |
| Box 401 |  |
| 405 30 Göteborg |  |
| Sweden |  |
|  |  |
|  |  |

|  |  |
| --- | --- |
|  | **Number of** |
| **Name and Address** |
| **Independent Ethics** | **Subjects** |
| **Committee (IEC)** | **Randomized** |
| Ethik-Kommission an der | 57 |
| Universität Regensburg |  |
| Franz-Josef-Strauss Allee |  |
| 11 |  |
| Regensburg 93042 |  |
| Germany |  |
|  |  |
|  |  |
| REK Nord | 107 |
| Universitetet i Tromsø |  |
| Det medisinske fakultet |  |
| 9037 Tromsø |  |
| Norway |  |
|  |  |
| Western Institutional | 4 |
| Review Board |  |
| 3535 Seventh Ave. SW |  |
| Olympia, WA 98502 |  |
|  |  |
| Tokyo Women’s Medical | 28 |
| University Hospital |  |
| Institutional Review |  |
| Board |  |
| 8-1 Kawada-cho |  |
| Shinjuku-ku Tokyo |  |
| Japan |  |
|  |  |
| Narita Red Cross Hospital | 53 |
| Institutional Review |  |
| Board |  |
| 90-1 Iida-cho Narita-shi |  |
| Chiba |  |
| Japan |  |
|  |  |
| Rakuwakai Otowa | 19 |
| Hospital Insutitutional |  |
| Review Board |  |
| Otowachinji-cho 2 |  |
| Yamashina-ku |  |
| Kyoto-shi Kyoto |  |
| Japan |  |
|  |  |

|  |  |
| --- | --- |
|  | **Number of** |
| **Name and Address** |
| **Independent Ethics** | **Subjects** |
| **Committee (IEC)** | **Randomized** |
| Keiwakai Oitaoka | 85 |
| Hospital Insutitutional |  |
| Review Board |  |
| 3-7-11,nishitsurusaki, |  |
| Oita-shi, Oita |  |
| Japan |  |
|  |  |
| Akane Family Clinic | 28 |
| Insutitutional Review |  |
| Board |  |
| 1-17-38,Akanedai,Aoba- |  |
| ku,Yokohama-city, |  |
| Kanagawa |  |
| Japan |  |
|  |  |
| Nagoya Daiichi Red | 107 |
| Cross Hospital |  |
| Institutional Review |  |
| Board |  |
| 3-35, Michishita-cho, |  |
| Nakamura-ku, Nagoya- |  |
| shi,Aichi |  |
| Japan |  |
|  |  |
| Nagoya Daini Red Cross | 96 |
| Hospital Institutional |  |
| Review Board |  |
| 2-9, Myoken-cho, Showa- |  |
| ku, Nagoya-shi, Aichi |  |
| Japan |  |
|  |  |
| Hiroshima City Hospital | 41 |
| Institutional Review |  |
| Board |  |
| 7-33,Motomachi, |  |
| Hiroshima-shi,Hiroshima |  |
| Japan |  |
|  |  |
| Penn State Milton S. | 15 |
| Hershey Medical Center |  |
| Human Subject |  |
| Protection Office Penn |  |
| State College of Medicine |  |
| 600 Centerview Dr. |  |
| Box 855 |  |
| Hershey, PA 17033 |  |
|  |  |


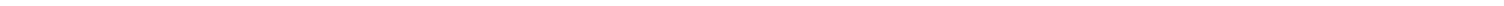


|  |  |
| --- | --- |
|  | **Number of** |
| **Name and Address** |
| **Independent Ethics** | **Subjects** |
| **Committee (IEC)** | **Randomized** |
| Western Institutional | 39 |
| Review Board |  |
| 3535 Seventh Ave. SW |  |
| Olympia, WA 98502 |  |
|  |  |
|  |  |
|  |  |
|  |  |
| Riverside Methodist | 17 |
| Hospital |  |
| Grady Memorial Hospital |  |
| IRB |  |
| 3545 Olentangy River |  |
| Rd., 4th Floor |  |
| Columbus, OH 43214 |  |
|  |  |
| New York Medical | 5 |
| College |  |
| Office Of Research |  |
| Administration |  |
| Administration Bldg. |  |
| Sunshine Cottage, 2nd |  |
| Floor |  |
| Valhalla, NY 10595 |  |
|  |  |
| REK Nord | 61 |
| Universitetet i Tromsø |  |
| Det medisinske fakultet |  |
| 9037 Tromsø |  |
| Norway |  |
|  |  |
| St. Joseph Hospital and | 5 |
| Medical Center |  |
| 350 W. Thomas Rd. |  |
| Phoenix, AZ 85013 |  |
|  |  |
| Western Institutional | 24 |
| Review Board |  |
| 3535 Seventh Ave. SW |  |
| Olympia, WA 98502 |  |
|  |  |


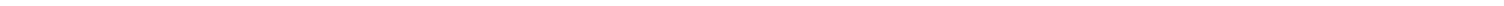


|  |  |
| --- | --- |
|  | **Number of** |
| **Name and Address** |
| **Independent Ethics** | **Subjects** |
| **Committee (IEC)** | **Randomized** |
| CEIC Comunidad | 113 |
| Autónoma del País Vasco |  |
| Edif.Dirección de |  |
| Farmacia C/ Donostia - |  |
| San Sebastián, 1 Vitoria, |  |
| Alava 01010 |  |
| Spain |  |
|  |  |
| Office of Research | 31 |
| Integrity |  |
| 315 Kinkead Hall |  |
| Lexington, KY 40536 |  |
|  |  |
| Pinehurst Medical Clinic | 19 |
| Sandhills Multi-IRB |  |
| Moore Regional Hosp |  |
| PO Box 3000 |  |
| Pinehurst, NC 28374 |  |
|  |  |
|  |  |
| HCA Richmond Division | 3 |
| Institutional Review |  |
| Board |  |
| 2621 Grove Avenue |  |
| Richmond, VA 23220 |  |
|  |  |
|  |  |
| Columbia University | 51 |
| Medical Center IRB |  |
| 722 West 168th Street |  |
| MSPH, Suite 426 |  |
| New York, NY 10032 |  |
|  |  |
| VA North Texas Health | 10 |
| Care System |  |
| Dallas VA Med Center |  |
| IRB |  |
| 4500 S. Lancaster Road |  |
| Mailcode 151 |  |
| Dallas, TX 75216 |  |
|  |  |


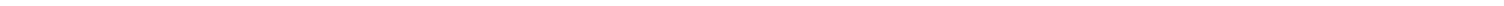

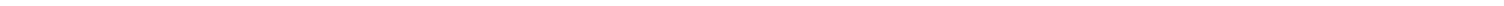

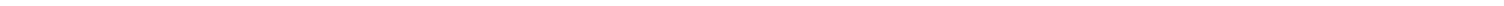


|  |  |
| --- | --- |
|  | **Number of** |
| **Name and Address** |
| **Independent Ethics** | **Subjects** |
| **Committee (IEC)** | **Randomized** |
| Seoul National University | 15 |
| Hospital IRB |  |
| 101 Daehangno |  |
| Jongno-gu, Seoul |  |
| 110-460 |  |
| Korea |  |
|  |  |
| Doylestown Hospital IRB | 13 |
| 595 West State Street |  |
| Doylestown, PA 18901 |  |
|  |  |
| VA Boston Healthcare | 12 |
| System (112) |  |
| Research & Development |  |
| 150 S. Huntington Ave., |  |
| Suite 151 |  |
| Jamaica Plain, MA 02130 |  |
|  |  |
| IUPUI and Clarian | 17 |
| Institutional Review |  |
| Boards |  |
| 620 Union Drive |  |
| Room 618 |  |
| Indianapolis, IN 46202 |  |
| Biomedical Research | 3 |
| Alliance of New York |  |
| 225 Community Drive |  |
| Suite 100 |  |
| Great Neck, NY 11021 |  |
|  |  |
| Oklahoma Cardiovascular | 18 |
| Surgeons |  |
| INTEGRIS Baptist |  |
| Medical Center Inc. IRB |  |
| 3300 NW Expressway |  |
| Oklahoma City, OK |  |
| 73112 |  |

|  |  |
| --- | --- |
|  | **Number of** |
| **Name and Address** |
| **Independent Ethics** | **Subjects** |
| **Committee (IEC)** | **Randomized** |
| Western Institutional | 26 |
| Review Board |  |
| 3535 Seventh Ave. SW |  |
| Olympia, WA 98502 |  |
|  |  |
| The Institutional Review | 10 |
| Board of the Tri-Service |  |
| General Hospital, |  |
| National Defense Medical |  |
| Center (TSGHIRB) |  |
| Tri-Service General |  |
| Hospital |  |
| No. 325, Sec. 2 |  |
| Cheng-Kung Road |  |
| Neihu 114, Taipei |  |
| Taiwan |  |
| Republic of China |  |
|  |  |
| Mackay Memorial | 17 |
| Hospital Institutional |  |
| Review Board |  |
| 8F, Administrative office, |  |
| Fu-Yin Building, No. 92, |  |
| Sec. 2, Chung-Shan North |  |
| Road |  |
| Taipei 104, Taiwan |  |
| Republic of China |  |
|  |  |
| IRB - Charles George | 22 |
| VAMC |  |
| Office of Research & |  |
| Development |  |
| 1100 Tunnel Road |  |
| Suite 213 |  |
| Asheville, NC 28805 |  |
|  |  |
| Western Institutional | 6 |
| Review Board |  |
| 3535 Seventh Ave. SW |  |
| Olympia, WA 98502 |  |
|  |  |
|  |  |
|  |  |


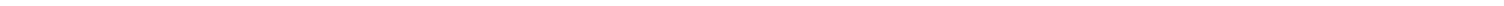


|  |  |
| --- | --- |
|  | **Number of** |
| **Name and Address** |
| **Independent Ethics** | **Subjects** |
| **Committee (IEC)** | **Randomized** |
| Human Research Office | 5 |
| U020 Learning Resource |  |
| Building |  |
| 2500 North State Street |  |
| Jackson, MS 39216 |  |
| Rochester General Health | 0 |
| Systems |  |
| Clinical Investigation |  |
| Committee |  |
| 1425 Portland Avenue |  |
| Rochester, NY 14621 |  |
|  |  |
| Alta Bates Summit | 18 |
| Institutional Review |  |
| Board |  |
| 2450 Ashby Avenue |  |
| Ashby, CA 94705 |  |
|  |  |
|  |  |
| Comitato Etico della | 4 |
| AUSL RM/D di Roma |  |
| Via di Casal Bernocchi, |  |
| 73 |  |
| Roma, 00125 |  |
| Italy |  |
|  |  |
| Western Institutional | 17 |
| Review Board |  |
| 3535 Seventh Ave. SW |  |
| Olympia, WA 98502 |  |
|  |  |
| Genesis Healthcare | 0 |
| System Institutional |  |
| Review Board |  |
| 800 Forest Avenue |  |
| Zanesville, OH 43701 |  |
|  |  |
|  |  |
|  |  |
| Medcenter One Health | 7 |
| Systems |  |
| Research and BioEthics |  |
| Committee |  |
| 300 N. 7th Street |  |
| Bismarck, ND 58501 |  |
|  |  |


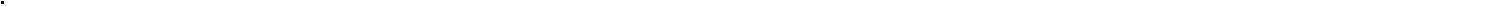

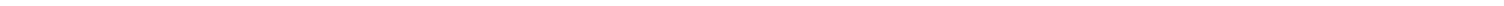

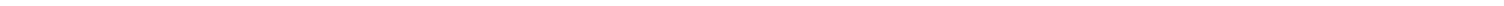


|  |  |
| --- | --- |
|  | **Number of** |
| **Name and Address** |
| **Independent Ethics** | **Subjects** |
| **Committee (IEC)** | **Randomized** |
| Comitê de Ética em | 53 |
| Pesquisa do |  |
| Instituto de Cardiologia |  |
| do RGS |  |
| Av. Princesa Isabel, 370 |  |
| Porto Alegre – RS |  |
| Brazil |  |
|  |  |
| Comité de Protection des | 21 |
| Personnes Ile de France |  |
| III |  |
| Hôpital Tarnier Cochin |  |
| 89, rue d’Assas |  |
| Paris 75006 |  |
| France |  |
|  |  |
|  |  |
| CEIC Comunidad | 146 |
| Autónoma del País Vasco |  |
| Edif.Dirección de |  |
| Farmacia C/ Donostia - |  |
| San Sebastián, 1 Vitoria, |  |
| Alava 01010 |  |
| Spain |  |
|  |  |
|  |  |
| CEIC Comunidad | 18 |
| Autónoma del País Vasco |  |
| Edif.Dirección de |  |
| Farmacia C/ Donostia - |  |
| San Sebastián, 1 Vitoria, |  |
| Alava 01010 |  |
| Spain |  |
|  |  |
|  |  |
| CEIC Comunidad | 89 |
| Autónoma del País Vasco |  |
| Edif.Dirección de |  |
| Farmacia C/ Donostia - |  |
| San Sebastián, 1 Vitoria, |  |
| Alava 01010 |  |
| Spain |  |
|  |  |
|  |  |


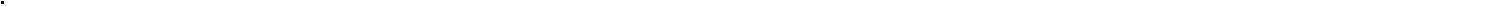

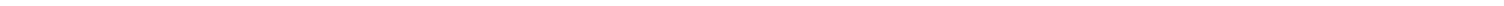


|  |  |
| --- | --- |
|  | **Number of** |
| **Name and Address** |
| **Independent Ethics** | **Subjects** |
| **Committee (IEC)** | **Randomized** |
| CEIC Comunidad | 3 |
| Autónoma del País Vasco |  |
| Edif.Dirección de |  |
| Farmacia C/ Donostia - |  |
| San Sebastián, 1 Vitoria, |  |
| Alava 01010 |  |
| Spain |  |
|  |  |
|  |  |
| Washington University | 22 |
| School of Medicine |  |
| Human Research |  |
| Protection Office (HRPO) |  |
| 660 S. Euclid Avenue |  |
| Box 8089 |  |
| St. Louis, MO 63110 |  |
|  |  |
| University Of Texas | 7 |
| Southwestern Medical |  |
| Center |  |
| 5323 Harry Hines Blvd. |  |
| Room C1.206/1st Floor |  |
| Dallas, TX 75390 |  |
|  |  |
| CEIC Comunidad | 0 |
| Autónoma del País Vasco |  |
| Edif.Dirección de |  |
| Farmacia C/ Donostia - |  |
| San Sebastián, 1 Vitoria, |  |
| Alava 01010 |  |
| Spain |  |
|  |  |
|  |  |
| Sanford Health IRB | 28 |
| 801 Broadway North |  |
| Fargo, ND 58122 |  |
|  |  |


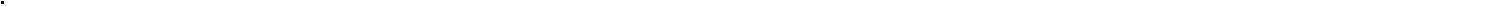

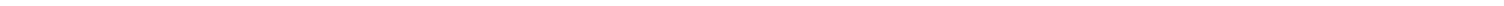


|  |  |
| --- | --- |
|  | **Number of** |
| **Name and Address** |
| **Independent Ethics** | **Subjects** |
| **Committee (IEC)** | **Randomized** |
| Saint Joseph’s Hospital of | 7 |
| Atlanta Institutional |  |
| Review Board |  |
| 5673 Peachtree |  |
| Dunwoody Road |  |
| Suite 650 |  |
| Atlanta, GA 30342 |  |
|  |  |
| St Anthony’s Hospital | 37 |
| 5666 E. State Street |  |
| Rockford, IL 61108 |  |
| Tokushukai group | 99 |
| Institutional Review |  |
| Board |  |
| 1-8-7, |  |
| Koujimachi,chiyoda- |  |
| ku,Tokyo |  |
| Japan |  |
|  |  |
|  |  |
| Tokushukai group | 27 |
| Institutional Review |  |
| Board |  |
| 1-8-7, |  |
| Koujimachi,chiyoda- |  |
| ku,Tokyo |  |
| Japan |  |
|  |  |
|  |  |
| Shizuoka Hospital | 38 |
| Institutional Review |  |
| Board |  |
| 10-93,Oute-machi,Aoi- |  |
| ku,Shizuoka- |  |
| city,Shizuoka |  |
| Japan |  |
|  |  |
|  |  |
|  |  |
| Western Institutional | 2 |
| Review Board |  |
| 3535 Seventh Ave. SW |  |
| Olympia, WA 98502 |  |
|  |  |
|  |  |
|  |  |
|  |  |


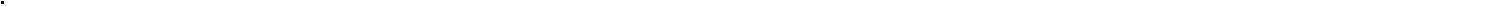

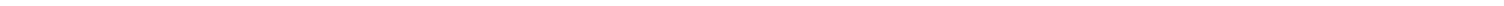

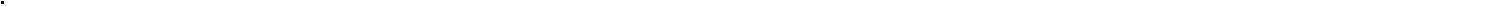


|  |  |
| --- | --- |
|  | **Number of** |
| **Name and Address** |
| **Independent Ethics** | **Subjects** |
| **Committee (IEC)** | **Randomized** |
| De Videnskabsetiske | 87 |
| Komitéer for Region |  |
| Hovedstaden - C |  |
| Regionsgården, Kongens |  |
| Vænge 2 Hillerød 3400 |  |
| Denmark |  |
|  |  |
| University of California, | 7 |
| Irvine Office of Research |  |
| Administration |  |
| 4199 Campus Drive |  |
| Suite 300 |  |
| Irvine, CA 92697 |  |
|  |  |
|  |  |
| Comite de Etica de Inv. | 54 |
| Clinica Fundacion |  |
| Cardioinfantil |  |
| Carrera 13 B # 163-85 |  |
| Piso 3 |  |
| Bogota |  |
| Colombia |  |
|  |  |
|  |  |
| Comite de Etica en Inv. | 28 |
| Fundacion Cardiovascular |  |
| de Colombia |  |
| Calle 155A # 23-58 |  |
| Piso 3 |  |
| Bucaramanga, Santander |  |
| Colombia |  |
|  |  |
| Comité de Ética en | 45 |
| Investigación |  |
| Independiente de Piura |  |
| Los Jacintos Mz.D Lt.6 |  |
| Urb. Miraflores Piura, |  |
| Peru |  |
|  |  |
| Hunter Homes McGuire | 12 |
| Veterans Affairs Medical |  |
| Center |  |
| McGuire IRB |  |
| 1201 Broad Rock Blvd |  |
| (151) |  |
| Richmond, VA 23249 |  |


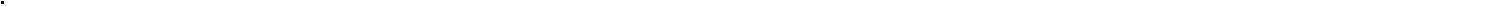

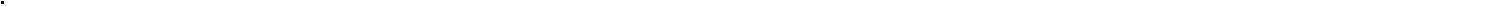


|  |  |
| --- | --- |
|  | **Number of** |
| **Name and Address** |
| **Independent Ethics** | **Subjects** |
| **Committee (IEC)** | **Randomized** |
| Western Institutional | 9 |
| Review Board |  |
| 3535 Seventh Ave. SW |  |
| Olympia, WA 98502 |  |
|  |  |
|  |  |
|  |  |
|  |  |
|  |  |
|  |  |
|  |  |
| Research Ethical and | 8 |
| Integral Clinic |  |
| Hospital Clinico |  |
| Universidad de Chile |  |
| Santos Dumont 999 |  |
| Santiago |  |
| Chile |  |
|  |  |
| ERC for Estearn | 11 |
| Metropolitan Puclic |  |
| Health Service Hospital |  |
| Salvador Av. Salvador |  |
| 364, Providencia |  |
| Santiago |  |
| Chile |  |
|  |  |
| Cork Clinical Research | 4 |
| Ethics Committee |  |
| Lancaster Hall |  |
| 6 Little Hanover Street |  |
| Cork |  |
| Ireland |  |
|  |  |
| Local Ethics Committee | 16 |
| of Federal State |  |
| Institution "Novosibirsk |  |
| Research Institute of |  |
| Circulation Pathology n.a. |  |
| Meshalkin of |  |
| Rosmedtechnologies" |  |
| 15, Rechkunovskaya |  |
| street, 630055, |  |
| Novosibirsk |  |
| Russian Federation |  |
|  |  |


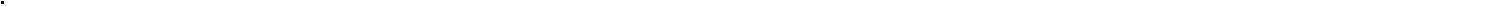


|  |  |
| --- | --- |
|  | **Number of** |
| **Name and Address** |
| **Independent Ethics** | **Subjects** |
| **Committee (IEC)** | **Randomized** |
| Local Ethics Committee | 12 |
| of Regional State |  |
| Institution of Health Care |  |
| "Novosibirsk Regional |  |
| Clinical Cardiology |  |
| Dispensary" |  |
| 6, Zalesskogo, build 8, |  |
| Novosibirsk 630047 |  |
| Russian Federation |  |
|  |  |
| Local Ethics Committee | 24 |
| of Federal State |  |
| Institution of Health Care |  |
| "Clinical Hospital #119 |  |
| of Federal Medical and |  |
| Biological Agency of |  |
| Russia" |  |
| Khimkinsky district, p.o. |  |
| Novogorsk, Moscow |  |
| 141435 |  |
| Russian Federation |  |
|  |  |
| Medical Research & | 16 |
| Ethics Commitee |  |
| Ministry of Health |  |
| Malaysia |  |
| c/o Institute for Health |  |
| Management |  |
| Jalan Rumah Sakit |  |
| Bangsar |  |
| 50900 Kuala Lumpur |  |
| Malaysia |  |
|  |  |
| IJN Ethics Committee | 6 |
| (IJNEC) |  |
| Institut Jantung Negara |  |
| 145 Jalan Tun Razak |  |
| 50400 Kuala Lumpur |  |
| Malaysia |  |
|  |  |
| Comite de Etica Instituto | 38 |
| Cardio-Neuro-Vascular |  |
| Corbic |  |
| 43A 18 Sur 135 Int. 331 |  |
| Sao Paulo Plaza |  |
| Antioquia, Medellin |  |
| 55420 |  |
| Colombia |  |
|  |  |

|  |  |
| --- | --- |
|  | **Number of** |
| **Name and Address** |
| **Independent Ethics** | **Subjects** |
| **Committee (IEC)** | **Randomized** |
| Comité d’Ethique | 10 |
| Hospitalo-Facultaire de |  |
| Liège |  |
| CHU Sart-Tilman B35 |  |
| B – 4000 Liège |  |
| Belgium |  |
|  |  |
| Research Comission of | 4 |
| Clinica Alemana |  |
| Av. Manquehue Norte |  |
| 1410 |  |
| Dp UCI Vitacura |  |
| Santiago 7650567 |  |
| Chile |  |
|  |  |
| Henry Ford Hospital | 0 |
| Henry Ford Health |  |
| System IRB |  |
| 2799 W. Grand Blvd. |  |
| CSP Basement |  |
| Detroit, MI 48202 |  |
|  |  |
| Patient Advocacy | 1 |
| Council, Inc. |  |
| 601 Bel Air Blvd. |  |
| Suite 315 |  |
| Mobile, AL 36606 |  |
|  |  |
| Northern X Regional | 23 |
| Ethics Committee |  |
| 3rd Floor, Unisys |  |
| Building |  |
| 650 Great South Road |  |
| Penrose, Auckland 1061 |  |
| New Zealand |  |
|  |  |
| Baystate Medical Center | 0 |
| Institutional Review |  |
| Board |  |
| 759 Chestnut Street |  |
| Springfield, MA 02116 |  |
|  |  |
|  |  |
|  |  |


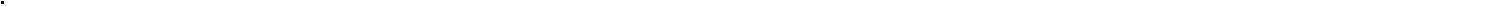

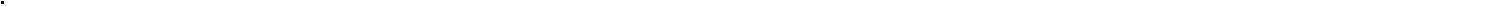


|  |  |
| --- | --- |
|  | **Number of** |
| **Name and Address** |
| **Independent Ethics** | **Subjects** |
| **Committee (IEC)** | **Randomized** |
| Palmetto Health IRB | 0 |
| 5 Richland Medical Park |  |
| Columbia, SC 29203 |  |
|  |  |
| Comite de Ética da | 22 |
| Faculdade de |  |
| Medicina/Universidade |  |
| de Brasília |  |
| Campus Universitário |  |
| Darcy Ribeiro - Asa |  |
| Norte |  |
| Brasília – DF |  |
| Brazil |  |
|  |  |
| SingHealth Centralized | 17 |
| Institution Review Board |  |
| Block A, 7 Hospital Drive, |  |
| #03-01 |  |
| Singapore Health Services |  |
| Pte Ltd. |  |
| SingHealth Research |  |
| Facilities |  |
| Singapore 169611 |  |
|  |  |
| St. Mark’s Hospital IRB | 10 |
| 1200 East 3900 South |  |
| Salt Lake City, UT 84124 |  |
|  |  |
| Western Institutional | 3 |
| Review Board |  |
| 3535 Seventh Ave. SW |  |
| Olympia, WA 98502 |  |

| Institutional Review | 12 |
| --- | --- |
| Board of Kyungpook |  |
| National University |  |
| Hospital |  |
| (130 Dongduk-ro) |  |
| 44-2 Samdeok-dong 2ga |  |
| Jung-gu, Daegu 700-721 |  |
| Korea |  |
|  |  |
| Comitê de Ética | 14 |
| HC/UFPR |  |
| R. General Carneiro, 181 |  |
| Curitiba – PR |  |
| Brazil |  |
| Hospital University de | 3 |
| Monterrey |  |
| Avenida Francisco |  |
| Madero esq. |  |
| Avenida Gonzalitos S/N |  |
| Monterrey, Nuevo León |  |
| 64460 |  |
| Mexico |  |
|  |  |
| Research & Development | 1 |
| Committee IRB |  |
| VA Medical Center |  |
| Washington, DC 20422 |  |
|  |  |
| Western Institutional | 6 |
| Review Board |  |
| 3535 Seventh Ave. SW |  |
| Olympia, WA 98502 |  |
|  |  |
| Genesis Health System | 0 |
| Institutional Review |  |
| Board |  |
| 1227 E. Rusholme Street |  |
| Davenport, IA 52803 |  |
|  |  |


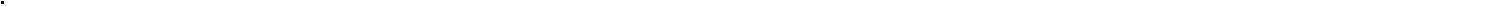

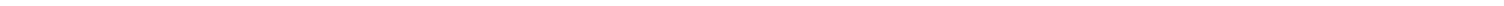


|  |  |
| --- | --- |
|  | **Number of** |
| **Name and Address** |
| **Independent Ethics** | **Subjects** |
| **Committee (IEC)** | **Randomized** |
| Baylor College of | 0 |
| Medicine & Affiliated |  |
| Hospitals Institutional |  |
| Review Board |  |
| 1 Baylor Plaza, 600D |  |
| Houston, TX 77030 |  |
|  |  |
| Ethik-Kommission an der | 8 |
| Universität Regensburg |  |
| Franz-Josef-Strauss Allee |  |
| 11 |  |
| Regensburg 93042 |  |
| Germany |  |
|  |  |
| Ethik-Kommission an der | 50 |
| Universität Regensburg |  |
| Franz-Josef-Strauss Allee |  |
| 11 |  |
| Regensburg 93042 |  |
| Germany |  |
|  |  |
| Western Institutional | 8 |
| Review Board |  |
| 3535 Seventh Ave. SW |  |
| Olympia, WA 98502 |  |
|  |  |
| Western Institutional | 5 |
| Review Board |  |
| 3535 Seventh Ave. SW |  |
| Olympia, WA 98502 |  |


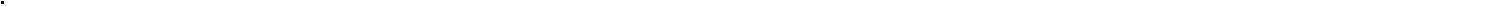

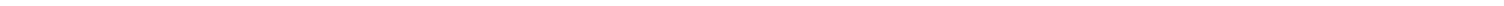


|  |  |
| --- | --- |
|  | **Number of** |
| **Name and Address** |
| **Independent Ethics** | **Subjects** |
| **Committee (IEC)** | **Randomized** |
| Comité d’éthique de la | 0 |
| recherche |  |
| 5000 Belanger |  |
| Montreal, Quebec |  |
| H1T 1C8 |  |
| Canada |  |
|  |  |
|  |  |
| Comité de Protection des | 65 |
| Personnes Ile de France |  |
| III |  |
| Hôpital Tarnier Cochin |  |
| 89, rue d’Assas |  |
| Paris 75006 |  |
| France |  |
|  |  |
| Local Ethics Committee | 11 |
| of Yaroslavl Regional |  |
| Clinical Hospital |  |
| 7, Yakovlevskaya str., |  |
| 150010 Yaroslavl |  |
| Russian Federation |  |
|  |  |
| Biomedical Ethics | 20 |
| Committee of State |  |
| Institution of Russian |  |
| Academy of Medical |  |
| Science “Scientific |  |
| Research Institution of |  |
| Cardiology Siberian |  |
| Branch of RAMS” |  |
| 111 A, Kievskaya str., |  |
| 634012, Tomsk, Russian |  |
| Federation |  |
|  |  |


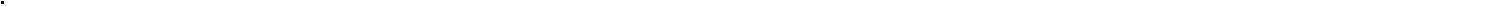

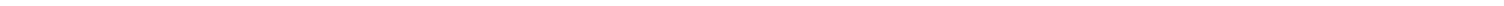

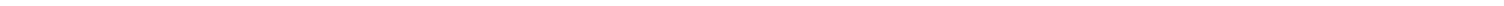


|  |  |
| --- | --- |
|  | **Number of** |
| **Name and Address** |
| **Independent Ethics** | **Subjects** |
| **Committee (IEC)** | **Randomized** |
| United Local Ethics | 12 |
| Committee of Municipal |  |
| Healthcare Institution |  |
| "Kemerovo Cardiology |  |
| Dispensary" and |  |
| Institution of Russian |  |
| Academy of Medical |  |
| Science "Heart and |  |
| Vessels Diseases |  |
| Complex Problems |  |
| Scientific Research |  |
| Institution of Siberian |  |
| Department of Russian |  |
| Academy of Medical |  |
| Science" |  |
| 6, Sosnovy Bulevard |  |
| 650002 Kemerovo |  |
| Russian Federation |  |
|  |  |
| Comité de Protection des | 15 |
| Personnes Ile de France |  |
| III |  |
| Hôpital Tarnier Cochin |  |
| 89, rue d’Assas |  |
| 75006 Paris |  |
| France |  |
|  |  |
| Cooper Health System | 0 |
| Institutional Review |  |
| Board |  |
| 3 Cooper Plaza |  |
| Camden, NJ 08103 |  |
| Comité de Protection des | 9 |
| Personnes Ile de France |  |
| III |  |
| Hôpital Tarnier Cochin |  |
| 89, rue d’Assas |  |
| Paris 75006 |  |
| France |  |
| Comité de Protection des | 3 |
| Personnes Ile de France |  |
| III |  |
| Hôpital Tarnier Cochin |  |
| 89, rue d’Assas |  |
| 75006 Paris |  |
| France |  |
| Lancaster General | 1 |
| Hospital Institutional |  |
| Review Board |  |
| 555 North Duke |  |
| Lancaster, PA 17602 |  |
| Abington Memorial | 5 |
| Hospital |  |
| 1200 Old York Road |  |
| Abington, PA 19001 |  |
| Northern X Regional | 0 |
| Ethics Committee |  |
| 3rd Floor, Unisys |  |
| Building |  |
| 650 Great South Road |  |
| Penrose, Auckland 1061 |  |
| New Zealand |  |
| Comité de Protection des | 9 |
| Personnes Ile de France |  |
| III |  |
| Hôpital Tarnier Cochin |  |
| 89, rue d’Assas |  |
| Paris 75006 |  |
| France |  |
| Comité de Protection des | 3 |
| Personnes Ile de France |  |
| III |  |
| Hôpital Tarnier Cochin |  |
| 89, rue d’Assas |  |
| 75006 Paris |  |
| France |  |
| Lancaster General | 1 |
| Hospital Institutional |  |
| Review Board |  |
| 555 North Duke |  |
| Lancaster, PA 17602 |  |
| Abington Memorial | 5 |
| Hospital |  |
| 1200 Old York Road |  |
| Abington, PA 19001 |  |
| Northern X Regional | 0 |
| Ethics Committee |  |
| 3rd Floor, Unisys |  |
| Building |  |
| 650 Great South Road |  |
| Penrose, Auckland 1061 |  |
| New Zealand |  |
| Cardiovascular Hospital | 0 |
| of Central Japan |  |
| Institutional Review |  |
| Board |  |
| 740, shimohakoda, |  |
| hokkitsu-machi, |  |
| shibukawa-shi, Gunma |  |
| Japan |  |
|  |  |


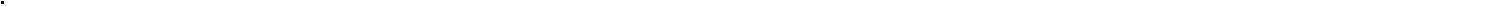


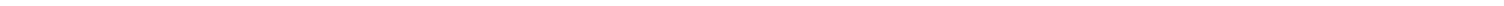

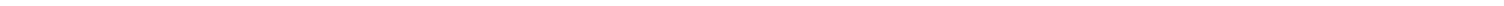


^*^If the original investigator has been replaced, that investigator’s name is followed by a slash (/) and the replacement investigator’s name is listed.

^†^Names of individual IEC members available upon request
